# Impact of the COVID-19 Pandemic on Pathological Stage, Tumor Characteristics and Surgical Outcomes of Oral Squamous Cell Carcinoma: A Retrospective Analysis

**DOI:** 10.3390/medicina61122225

**Published:** 2025-12-17

**Authors:** Samer Omari, Murad AbdelRaziq, Kutaiba Alkeesh, Alaa Muhana, Imad Abu El-Naaj, Yasmin Ghantous

**Affiliations:** 1 Department of Oral and Maxillofacial Surgery, Baruch Padeh Medical Center Poriya, Tiberies 20200, Israel; 2 The Azrieli Faculty of Medicine, Bar Illan University, Tel Aviv 20200, Israel

**Keywords:** oral squamous cell carcinoma, COVID-19 pandemic, cancer diagnosis delay, pathological staging, TNM, depth of invasion, surgical outcomes, head and neck cancer

## Abstract

*Background and Objectives*: Oral Squamous Cell Carcinoma (OSCC) requires early diagnosis for favorable outcomes, but global healthcare disruptions caused by the COVID-19 pandemic severely affected cancer care delivery. This study aimed to investigate the pandemic’s influence on OSCC pathological staging and disease-related characteristics at a single medical center. *Materials and Methods*: A retrospective study was conducted on 148 patients who underwent curative-intent surgery for newly diagnosed OSCC between March 2018 and October 2024. Patients were stratified into a Pre-COVID-19 group (March 2018–January 2020, N = 52) and a Post-COVID-19 group (February 2020–October 2024, N = 96). Patient demographics and risk factors were compared using Chi-squared and Wilcoxon rank-sum tests, while pathological stage, Depth of Invasion (DOI), and surgical outcomes were analyzed. *Results*: Patient demographics, risk factors, and comorbidities were comparable between the two groups. The Post-COVID-19 cohort presented with significantly more advanced pathological disease, evidenced by an increase in overall TNM stage, including a dramatically higher rate of Stage 4 diagnosis (35% vs. 3.2% in the Pre-COVID-19 group). This group also showed a significantly higher Depth of Invasion (median DOI: 5.0 mm vs. 3.0 mm). Consequently, the Post-COVID-19 group required more aggressive treatment (e.g., higher rates of adjuvant radiotherapy) and experienced worse short-term outcomes, including significantly longer hospitalization (median 15 days vs. 6 days) and higher rates of postoperative pulmonary infection and tracheostomy. *Conclusions*: The COVID-19 pandemic was associated with a dramatic shift toward the diagnosis of OSCC at advanced pathological stages. This diagnostic delay necessitated more complex surgical management and resulted in significantly worse short-term outcomes. These findings underscore the urgent need for resilient strategies to prevent systemic diagnostic delays during public health crises.

## 1. Introduction

Oral squamous cell carcinoma (OSCC) represents the most common malignancy of the oral cavity, with a worldwide incidence that continues to rise [1]. Although historically associated with traditional risk factors like tobacco and alcohol use, a notable epidemiological shift is occurring, with an increasing number of cases in younger, non-smoking populations [2]. Llewellyn showed that the majority of patients younger than 40 years had no exposure to conventional risk factors [3]. The benign HPV-related lesions of the oral mucosa recognized by the World Health Organization are squamous cell papilloma (SP), condyloma acuminatum (CA), verruca vulgaris (VV), and focal epithelial hyperplasia (FEH) [2]. While the link is certain for oropharyngeal cancer, this trend highlights the emerging, though still potential, association of Human Papilloma Virus (HPV) in the etiology of OSCC [4,5,6].

Clinical and histological examinations remain the gold standard for diagnosing OSCC, yet screening for early identification, developing new diagnostic methods for early detection, and receiving timely intervention are crucial for achieving better outcomes. Several novel techniques have been proposed and documented in recent years, including staining methods, imaging and optical spectroscopy, reflectance confocal microscopy (RCM), liquid biopsy, microarray, nanotechnology, salivary biomarkers, and AI-based screening and detection [7,8,9,10]. The potential role of these methods remains no substitute for tissue biopsy and histopathological examination [11].

The significant morbidity and high mortality rates associated with OSCC underscore the critical importance of early diagnosis and timely, definitive treatment, which is primarily achieved via surgical resection guided by the AJCC/UICC 8th edition staging system [12,13]. Surgical cure, adequate neck management, and modern soft tissue and bone reconstruction techniques are considered the ideal treatment modality for nonmetastatic, resectable OSCC, whereas (chemo) radiotherapy can be used in cases where primary surgery is not feasible or as adjuvant therapy in certain cases of advanced disease [14].

Despite the established link between early-stage disease and improved patient outcomes, a substantial number of patients are still diagnosed at advanced stages, leading to significant functional disability and a poor prognosis [15,16]. This issue was further complicated by the global COVID-19 pandemic, which caused unprecedented disruption to healthcare services. National lockdowns, the reallocation of medical resources, and a sharp decline in in-person visits resulted in widespread delays in the referral, diagnosis, and treatment of cancer patients [17,18]. Xu et al. reported a substantial reduction in inpatient, emergency department, and outpatient utilization, attributed to delays in elective care during COVID-19 restrictions [19].

Crucially, these provider- and system-level delays were compounded by significant alterations in patient behavior and public awareness, which are also pertinent to diagnostic delays. Fear of contracting the SARS-CoV-2 virus in clinical settings, coupled with public health messaging prioritizing pandemic safety, led many individuals to intentionally delay or forego seeking medical attention for non-COVID symptoms, including oral lesions [20]. Furthermore, the intense global focus on COVID-19 symptomatology may have inadvertently reduced general public awareness and knowledge of the early signs of high-risk conditions such as OSCC [20], contributing to longer patient-mediated delays between symptom onset and initial consultation. This combination of systemic constraints and patient-driven avoidance created a perfect storm for upstaging and advanced presentation of malignancies.

In addition to systemic delays, research suggests a potential increase in peri- and postoperative morbidity following SARS-CoV-2 infection, particularly involving pulmonary pathology. Patients undergoing major head and neck surgery, already at high risk for aspiration and pulmonary complications, may face compounded risks due to pre-existing or residual inflammatory effects from the virus. Cancer patients are particularly vulnerable to SARS-CoV-2 infection and development of more severe symptoms [21]. These patients are more likely to develop severe infection and complications, including acute respiratory distress syndrome (ARDS), septic shock, and multiorgan failure leading to death [22].

Furthermore, research indicates that SARS-CoV-2 may also exert direct biological effects on tumor progression by activating oncogenic pathways and generating a chronic pro-inflammatory microenvironment. Norouzi et al. discussed the role of COVID-19 in patients with multiple cancers and showed that it adversely affected the progression, prognosis, and response to chemotherapy regimens [23]. Zalpoor et al. pointed out that autophagy induced by the virus can lead to cancer progression, chemo-resistance and recurrence through the activation of key signaling pathways leading to upregulation of various factors [24]. In another study, Saini et al. suggested that COVID-19 predisposes recovered patients to cancer development by modulating oncogenic pathways and promoting chronic low-grade inflammation and tissue damage [25]. Gopalakrishnan et al. discussed the role of Angiotensin II in the carcinogenesis and progression of head and neck squamous cell carcinoma (HNSCC), as a pro-tumoral agent that promotes angiogenesis, proliferation, and tumor invasion, and facilitates metastasis of cancer cells. COVID-19 was found to reduce the availability of angiotensin-converting enzyme 2 (ACE2) receptors, which causes upregulation of angiotensin II, and so it aids in the progression of the disease [26].

A decreased interval between tumor-related symptoms and diagnosis of OSCC favorably influences tumor stage at diagnosis, subsequent treatment outcomes, survival rates and quality of life. Considering that treatment is most effective in early stages, the impact of late diagnosis was demonstrated in several studies by higher morbidity and mortality rate among these patients [27,28,29]. Given the invasive nature of OSCC surgery, which often requires tracheostomy and prolonged recovery, it is crucial to investigate whether the post-COVID-19 period is associated not only with advanced disease but also with a distinct pattern of postoperative complications, especially those affecting respiratory function.

Given these challenges, our study aims to investigate the impact of the COVID-19 pandemic on the pathological stage and the management of patients with OSCC who underwent curative-intent surgery. We hypothesize that the presenting pathological TNM stage of patients at our institution has been upgraded following the outbreak of the pandemic in February 2020. By analyzing and comparing the clinical and pathological features of patients treated before and after this period, we seek to comprehensively evaluate the pandemic’s sequelae and its influence on OSCC patient populations.

## 2. Materials and Methods

### 2.1. Study Design and Patient Cohort

This study was designed to compare Oral Cancer patients before and after February 2020, when the state of health emergency was declared in Israel in terms of the COVID-19 pandemic. The aim was to compare the patient, professional, and treatment delays, as well as the patient outcomes, between a pre-pandemic (Pre-COVID) cohort and a post-pandemic cohort (Post-COVID).

Delay Definitions:

1. Patient Delay: The time interval between the patient’s first awareness of symptoms (e.g., persistent lump, non-healing ulcer) and the first consultation with any healthcare professional (HCP) (e.g., general practitioner or dentist) regarding the symptom.
2. Professional Delay (or Diagnostic Delay): The time interval from the first consultation with any HCP regarding the symptom to the date of the confirmed histopathological diagnosis of OSCC.
3. Treatment Delay: The time interval from the date of the confirmed histopathological diagnosis of OSCC to the date of the initiation of definitive curative-intent surgery.

Data used to calculate these intervals were extracted from the patient’s electronic medical record through structured interviews and documentation logs.

Retrospective analysis of all patients who were diagnosed with histopathologically confirmed OSCC whose primary definitive treatment was curative-intent surgical resection at the Department of Oral and Maxillofacial Surgery at Tzafon Medical Center, Poria, Israel, with approval from the hospital’s Helsinki Ethics Committee (0083-24-POR).

Tzafon Medical Center is a major regional referral center serving a diverse patient population from northern Israel [13]. The center maintains a comprehensive, multidisciplinary Head and Neck Oncology service, ensuring patients receive standardized care involving specialized surgical oncology, reconstruction, and dedicated medical and radiation oncology support. This comprehensive capability ensures that the reported treatment intensity reflects the disease’s complexity rather than a lack of institutional resources.

Patients who presented with a confirmed primary diagnosis of Oral Squamous Cell Carcinoma (OSCC) by histopathological examination between March 2018 and January 2020 were allocated to the Pre-COVID group, while patients diagnosed between February 2020 and October 2024 were allocated to the Post-COVID group. The exclusion criteria were patients with a non-malignant diagnosis or consulting for an oral cancer recurrence; adult patients (age 1 years at diagnosis); and patients for whom the medical records were incomplete. Specifically, patients with missing data for any of the primary variables of interest (pathological TNM stage, survival status, or time-to-treatment) were excluded; thus, a complete-case analysis (listwise deletion) was performed. All patients were staged at their initial presentation using the American Joint Committee on Cancer (AJCC) 8th edition staging system. It is noted that a systematic history of SARS-CoV-2 infection status was not consistently documented or available for the full retrospective cohort and was therefore not included as a variable in the primary analysis.

### 2.2. Data Collection

Demographic, clinical, and histopathological data were extracted from the hospital’s electronic medical record system, and reports from the Department of Pathology confirmed histopathological diagnoses. Patients were stratified into two groups based on their date of diagnosis, as mentioned above, while February 2020 was chosen as the cut-off point, corresponding to the initial widespread outbreak and first national restrictions in Israel.

### 2.3. Variables of Interest

The primary variable of interest was the pathological TNM stage at presentation. Other collected data included information across four key domains:

- Sociodemographic and Risk Factors: Age, sex, tobacco use, and alcohol use.
- Clinical and Pathological Characteristics: Diagnosis, comorbidities (e.g., chronic obstructive pulmonary disease and diabetes mellitus), pathological tumor size (T-stage), nodal status (number of involved lymph nodes and N-stage), overall Tumor–Node–Metastasis (TNM) staging classification (AJCC 8th edition), Depth of Invasion (DOI), presence of Lymphovascular Invasion (LVI), and presence of Perineural Invasion (PNI).
- Treatment Details: Date and type of primary treatment.
- Treatment and Outcomes: Type of surgical therapy, use of neck dissection, administration of adjuvant therapy (radiotherapy/chemotherapy), total hospitalization duration, ICU length of stay, and the occurrence of postoperative complications, including pneumonia and prolonged tracheostomy.

Postoperative Complication Criteria:

- Postoperative Pulmonary Infection (POP): A diagnosis of POP was retrospectively confirmed if the patient record contained all three of the following criteria documented within the initial hospitalization period: (1) Clinical documentation of fever (≥38 °C) or leukocytosis (WBC > 10 × 10^9^ L) or purulent sputum; (2) New or progressive infiltrate confirmed via Chest X-ray or CT scan; and (3) Initiation of an appropriate course of systemic antibiotics specifically for treating pneumonia.
- Prolonged Tracheostomy: Tracheostomy was considered prolonged if the cannula remained in situ for a period greater than 7 days postoperatively, or if decannulation failed during the initial hospitalization period, necessitating discharge with the tracheostomy in situ.

### 2.4. Statistical Analysis

Data were compiled using Microsoft Excel and analyzed with RStudio^®^ (version 2021.09.0 Build 351). Descriptive statistics were used to summarize patient information.

The assumption of normality for all continuous variables was tested using the Shapiro–Wilk test and visual inspection of Q-Q plots. Based on this assessment, continuous variables with a normal distribution were presented as means (Standard Deviation, SD) and compared using the independent samples *t*-test (parametric). Continuous variables that were not normally distributed were presented as medians (25–75% interquartile range) and compared using the Wilcoxon rank-sum test (nonparametric).

Categorical variables were presented as frequencies and percentages (%) and compared using the Chi-squared test or Fisher’s exact test, as appropriate (Fisher’s exact test was used when more than 20% of the cells had expected counts less than 5). A difference with a *p*-value less than 0.05 was considered statistically significant.

Short-Term Outcome Justification: Short-term outcomes, including total hospitalization duration, ICU length of stay, and the occurrence of acute postoperative complications (e.g., pneumonia and prolonged tracheostomy), are not influenced by the duration of patient follow-up. These metrics capture events occurring during the initial treatment and inpatient recovery period, ensuring comparability across subsequent patient monitoring periods.

#### 2.4.1. Sample Size Estimation and Validation

As this was a retrospective study utilizing a fixed, non-interventional cohort, a formal a priori sample size calculation was not feasible. However, to validate the statistical efficacy of our cohort size, we performed a retrospective sample size estimation procedure based on the primary outcome: the difference in the incidence rate of Stage IV OSCC.

We defined the minimum clinically meaningful difference (MCMD) in Stage IV incidence between the Pre-COVID and Post-COVID cohorts as a 15% absolute increase (i.e., from the baseline 3.2% to 18.2%). Assuming a statistical power of 80% and a two-sided alpha level of 0.05, a standard sample size calculation (using P_1_ = 0.032 and P_2_ = 0.182) requires approximately N = 120 total patients.

Our final cohort size of N = 148 substantially exceeds this required threshold, confirming that the study was adequately powered to detect a clinically meaningful difference in advanced-stage disease. Consequently, the actual observed difference of 31.8% was detected with extremely high power (beta > 0.99).

#### 2.4.2. Time-to-Event Analysis

Analyses of recurrence patterns and survival outcomes during the intermediate follow-up period were conducted using the Kaplan–Meier method to account for the substantial differences in follow-up duration between the cohorts. Overall Survival (OS) and Disease-Free Survival (DFS) were calculated from the date of surgery. Survival curves were compared using the Log-rank test, and data were censored at the last documented follow-up visit or at the study end date (October 2024). Intermediate outcomes (e.g., 2-year OS and DFS rates) are reported.

## 3. Results

This study included a total of 148 histopathologically diagnosed OSCC cases, and operated over a 79-month period, with a mean age of 66 years. Fifty-two cases (35.1%) were in the Pre-COVID-19 group, and 96 cases (64.9%) were in the Post-COVID-19 group. The median age at diagnosis in the study cohort, the post-COVID and pre-COVID groups, was 66, 67, and 67 years, respectively. There were no statistically significant differences between the two groups in patient demographics, including age (*p* = 0.34) and gender (*p* = 0.13). Similarly, the distribution of major risk factors such as smoking habits (*p* = 0.67), alcohol use (*p* = 0.9) and pre-existing medical comorbidities (COPD, *p* = 0.33; DM, *p* = 0.19) was comparable between the Pre- and Post-COVID-19 groups (Table 1).

On the other hand, significant differences were found regarding tumor characteristics (Table 2). The Pre-COVID-19 group showed a higher prevalence of oral tongue OSCC (45% vs. 33%), while the Post-COVID-19 group had a significantly higher proportion of tumors in the lower and upper alveolus (35% vs. 6.5%).

Furthermore, the Post-COVID-19 group presented with significantly more advanced T stages (*p* = 0.002). A notably higher percentage of patients in the Post-COVID-19 group were classified as T4 (27% vs. 0%) at presentation. Correspondingly, the mean Depth of Invasion (DOI) was significantly higher in the Post-COVID-19 group (7.1 mm vs. 4.0 2.2 mm, *p* = 0.044). Also, the overall pathological TNM stage was significantly more advanced in the Post-COVID-19 group (*p* = 0.00). Patients in the Post-COVID-19 group were diagnosed with Stage 4 disease at a rate more than ten times higher than the Pre-COVID-19 group (35% vs. 3.2%).

### Treatment and Outcome Measures

The advanced disease status in the Post-COVID-19 group correlated with more aggressive treatment and worse short-term outcomes (Table 3):

- Adjuvant Therapy: The need for post-surgery Adjuvant Radiotherapy was significantly higher in the Post-COVID-19 group (39% vs. 13%, *p* = 0.014), indicating a higher burden of advanced, higher-risk disease. Also, a substantially greater proportion of patients in the Post-COVID-19 group required a neck dissection (88% vs. 45%, *p* < 0.001).
- Hospitalization: The total hospitalization period was significantly more extended in the Post-COVID-19 group (median 15 days vs. 6 days, *p* < 0.001). This was also true for the ICU length of stay (median 2 days vs. 0 days, *p* < 0.001).
- Complications: Postoperative pulmonary infection (POP) and the need for prolonged tracheostomy were observed significantly more frequently in the Post-COVID-19 group (*p* = 0.013 and *p* < 0.001, respectively).

## 4. Discussion

The findings from this retrospective study indicate a significant escalation in the pathological stage and severity of OSCC diagnosed during the COVID-19 pandemic era. The shift to significantly more advanced cancer staging at diagnosis (Stage III and IV), with the proportion of patients presenting with Stage IV disease being over ten times higher in the Post-COVID-19 group (35% vs. 3.2%), directly supports the hypothesis that the pandemic contributed to a critical delay in diagnosis.

### 4.1. Comparative Analysis with Other Head and Neck Malignancies

A systematic evaluation of other HNSCC subsites is necessary to isolate the unique factors driving upstaging [30,31,32,33]. Oral SCC presents a distinctive challenge: the lesion is often visible to the patient or accessible by a dentist, making its diagnosis highly reliant on routine professional surveillance. Our findings reflect the acute impact of the cessation of routine dental check-ups (professional screening delay) and patient fear [34], which eliminated this primary detection filter. This contrasts sharply with delays seen in subsites like laryngeal carcinoma, where studies often cite diagnostic delay due to non-specific symptoms (e.g., hoarseness) being misattributed to common infections like COVID-19 [35,36,37]. Similarly, nasopharyngeal carcinoma often suffered from systemic delay due to the disruption of specialized diagnostic pathways. Recognizing these distinctions is crucial: while all HNSCC sites suffered, our OSCC cohort was uniquely penalized by the failure of the routine screening pathway. Our local findings of a significant increase in T stage (*p* = 0.002) and Depth of Invasion (DOI) (*p* = 0.044) are consistent with this global consensus, suggesting that systemic and behavioral delays allowed early-stage tumors to progress locally before patients sought or received definitive diagnosis.

### 4.2. Analysis of Delay Components

The severe upstaging observed in our cohort is not attributable to a single factor but rather to the combined effects of the Corona Collateral Damage Syndrome (CCDS), a syndrome encompassing indirect health consequences of COVID-19, characterized by delayed diagnosis and treatment of non-COVID-19 conditions and subsequent adverse outcomes attributable to pandemic-related disruptions [38,39,40]. This syndrome was driven by intersecting delay components: Patient-Related Delay (widespread fear of contracting SARS-CoV-2), Professional-Related Delay (reduction in non-urgent visits to dental/primary care offices), and System-Related Delay (hospital resource diversion necessitating longer surgical wait times). It is the synergistic combination of these patient-, professional-, and system-level delays that overwhelmingly accounts for the observed shift to advanced OSCC staging.

The observed shift in anatomical distribution, marked by a significant increase in alveolar tumor prevalence (35% vs. 6.5%) concurrent with decreased tongue tumor incidence (33% vs. 45%), warrants specific mechanistic explanation. This shift likely reflects how pandemic restrictions differentially impacted the lesion detection pathways. Alveolar ridge tumors are often initially asymptomatic and heavily reliant on detection via routine dental screening [40]. The near-cessation of routine dental care during lockdowns eliminated this critical professional filter, suggesting that the impact of professional delay was most acutely felt in anatomically less conspicuous regions.

### 4.3. Tumor Invasiveness and Clinical Outcomes

The progression to a more advanced stage directly correlated with increased treatment intensity and significantly poorer short-term outcomes. The clinical relevance of this progression is reinforced by enhanced pathological analysis. The significant increase in DOI (*p* = 0.044) serves as the primary histological marker of increased aggression. Furthermore, the higher incidence of Adjuvant Radiotherapy (39% vs. 13%) serves as a clinical surrogate for high-risk pathology (including the likely presence of LVI and PNI), which mandates intensive postoperative treatment. The significantly more extended hospitalization periods (median 15 days vs. 6 days) and higher rates of postoperative pulmonary infection (POP) and prolonged tracheostomy emphasize the greater complexity and morbidity.

While the delay mechanism is the primary driver of upstaging, we note the emerging, yet speculative, evidence regarding the direct biological effects of SARS-CoV-2. Some literature suggests the virus may utilize multiple strategies to activate oncogenic pathways and induce a pro-inflammatory microenvironment [41,42], which could potentially contribute to more aggressive tumor behavior or a predisposition to postoperative pulmonary morbidity [43]. However, as our study design is retrospective and lacks systematic data on prior SARS-CoV-2 infection status, the evidence overwhelmingly favors diagnostic and treatment delay as the principal cause of the observed clinical deterioration.

### 4.4. Operational Refinement: Actionable Protocols for Disaster Preparedness

To ensure the findings from this study translate into meaningful clinical practice, the proposed disaster preparedness strategy must be transformed into concrete, actionable protocols. We recommend the following specific implementations:

1. Prioritized OSCC Screening Pathways: Implement a tier-based triage protocol for suspected OSCC, allowing immediate specialist referral for patients presenting with non-healing oral ulcers or red/white lesions persisting beyond two weeks. This mitigates the professional-level diagnostic delay.
2. Telemedicine for Diagnostics and Monitoring: Establish secure, high-resolution telemedicine platforms (e.g., teledentistry) for remote assessment of suspicious oral lesions. These platforms should also be utilized for non-critical postoperative monitoring to enhance resource conservation and address Patient Delay.
3. Early Warning Systems for Postoperative Complications: Develop and implement an enhanced Post-Surgical Early Warning System (EWS) in the postoperative period. This EWS should include specific protocols for intensified respiratory therapy and early tracheostomy weaning assessment, directly targeting the high rates of morbidity (POP, prolonged tracheostomy) observed in the Post-COVID cohort.

Furthermore, empirical studies highlight the efficacy of implementing rapid-access “one-stop” diagnostic clinics for suspected HNSCC [36] and the creation of dedicated surgical oncology hubs to ensure the continuity of curative-intent procedures [38]. Implementing these concrete measures would substantially improve the clinical applicability and translational potential of this research.

## 5. Conclusions

The COVID-19 pandemic was associated with a dramatic shift toward diagnosing OSCC at significantly advanced pathological stages at our institution, resulting in more complex treatment, higher rates of adjuvant therapy, and worse short-term surgical outcomes.

The data definitively show that this clinical deterioration was primarily driven by the confluence of patient-, professional-, and system-related diagnostic delays. While the potential biological role of SARS-CoV-2 in cancer progression warrants ongoing investigation, the overwhelming evidence from our cohort points to the delay mechanisms as the major cause of upstaging.

These findings underscore the critical need for strategies to mitigate pandemic-related delays and improve early-stage detection. Moving forward, these results highlight the imperative to integrate robust, actionable pandemic preparedness strategies that safeguard non-emergency oncological screening and referral pathways, thereby ensuring the continuity of early cancer detection and minimizing adverse intermediate-term prognostic outcomes during future health crises.

## Figures and Tables

**Table 1:** Patient’s demographic data.

Characteristic	Overall, N = 148	POST, N = 96 *^1^*	PRE, N = 52 *^1^*	*p*-Value
Age at Diagnosis	66 (54–74)	65 (52–74)	67 (54–81)	*0.34*
Gender				*0.13*
Female	59 (40%)	44 (46%)	15 (29%)	
Male	89 (60%)	52 (54%)	37 (71%)	
Smoking Habits	78 (53%)	49 (51%)	30 (58%)	*0.67*
Alcohol use	5 (3.4%)	3 (3.5%)	1 (3%)	*0.9*
DM (Diabetes Mellitus)	33 (22%)	25 (26%)	8 (14%)	0.19
COPD (Chronic Obstructive Pulmonary Disease)	9 (5.8%)	4 (3.5%)	5 (10%)	0.33

*^1^* Median (25–75%)/Mean (SD); n/N (%).

**Table 2:** Tumor characteristics.

Characteristic	Overall, N = 148 *^1^*	POST, N = 96 *^1^*	PRE, N = 52 *^1^*	*p*-Value
Tumor sub-site				0.003
Oral tongue	56 (38%)	32 (33%)	23 (45%)	
Lip	27 (18%)	8 (8.8%)	19 (35%)	
Flour of Mouth	13 (9.1%)	11 (11%)	2 (6.5%)	
Buccal mucosa	8 (5.7%)	5 (5.3%)	3 (6.5%)	
lower + upper alveolus	37 (25%)	33 (35%)	4 (6.5%)	
Retromolar	5 (3.4%)	5 (5.3%)	0 (0%)	
Palate	2 (1.1%)	2 (1.8%)	0 (0%)	
Pathological T stage				0.002
T1	65 (44%)	33 (34%)	32 (61%)	
T2	44 (30%)	28 (29%)	16 (32%)	
T3	14 (9.2%)	11 (11%)	3 (6.5%)	
T4	25 (17%)	25 (27%)	0 (0%)	
Pathological N stage				0.17
N0	118 (80%)	70 (73%)	48 (94%)	
N1	10 (6.9%)	8 (8.9%)	2 (3.2%)	
N2	15 (10%)	13 (14%)	2 (3.2%)	
N3	3 (2.3%)	3 (3.6%)	0 (0%)	
Neck Dissection	108 (73%)	84 (88%)	23 (45%)	<0.001
DOI (Depth of Invasion)—Mean ± SD	5.9 ± 5.3	7.1 ± 6.2	4.0 ± 2.2	0.044
8th Edition AJCC Pathological TNM stage				0.001
I	59 (40%)	27 (28%)	32 (61%)	
II	35 (24%)	22 (23%)	13 (26%)	
III	18 (12%)	13 (14%)	5 (9.7%)	
IV	36 (24%)	34 (35%)	2 (3.2%)	

*^1^* n/N (%); Median (25–75%)/Mean (SD).

**Table 3:** Other Parameters.

Characteristic	Overall, N = 148 *^1^*	POST, N = 96 *^1^*	PRE, N = 52 *^1^*	*p*-Value *^2^*
POP (Post-operative pulmonary infection)	18 (12%)	16 (16%)	2 (3%)	0.013
Tracheostomy	77 (52%)	71 (74%)	6 (10%)	<0.001
Primary Surgical Therapy	148 (100%)	96 (100%)	52 (100%)	
Primary chemotherapy	0	0	0	
Primary radiation Therapy	0	0	0	
Post Surgery Chemotherapy	16 (11%)	15 (16%)	1 (2%)	0.15
Post Surgery Radiotherapy	44 (30%)	37 (39%)	7 (13%)	0.014
Pt presented with recurrence	8 (5.7%)	3 (3.5%)	5 (10%)	0.33

*^1^* n/N (%); *^2^* Fisher’s exact test; Pearson’s Chi-squared test.

## Data Availability

The data presented in this study are available on request from the corresponding author due to the privacy of the patients included in the study, since sensitive data is included.
